# Supplementary material for: Ecological factors associated with persistent circulation of multiple highly pathogenic avian influenza viruses among poultry farms in Taiwan during 2015-17
Source: PLoS One. 2020 Aug 13;15(8):e0236581. doi: 10.1371/journal.pone.0236581 (PMC7425926; doi:10.1371/journal.pone.0236581)
Supplement: S1 Table — (DOCX) [file pone.0236581.s001.docx]

Table S1.Univariate logistic regression modeling results after stepwise selection comparing the hot zone and non-hot zone areas of HPAIV-confirmed outbreak farms based on 3km local spatial clustering analysis during 2015-2017, Taiwan

|  | 2015 | | | | 2016 | | | | 2017 | | | |
| --- | --- | --- | --- | --- | --- | --- | --- | --- | --- | --- | --- | --- |
|  | Estimate | OR^#^ | 95% CI^$^ | p-value | Estimate | OR | 95% CI | p-value | Estimate | OR | 95% CI | p-value |
| nrwaterD |  |  |  |  |  |  |  |  |  |  |  |  |
| medium | 2.19 | 8.94 | 3.85-20.75 | <0.001*** | 18.03 | 67895000 | 3.1529 x10^-66^ - NA | 0.992 | 2.80 | 16.52 | 5.97-49.70 | <0.001*** |
| high | 3.39 | 29.73 | 16.57-58.22 | <0.001*** | 18.75 | 139280000 | 6.4682 x10^-8^ - NA | 0.992 | 3.42 | 30.47 | 13.97-80.13 | <0.001*** |
| allrD |  |  |  |  |  |  |  |  |  |  |  |  |
| medium | 2.21 | 9.14 | 2.99-39.72 | <0.001*** | 17.58 | 43100000 | 7.8084 x10^-82^ - NA | 0.993 | 2.27 | 9.66 | 2.57-62.70 | 0.00332 ** |
| high | 4.37 | 78.83 | 29.22-323.11 | <0.001*** | 18.47 | 104540000 | 1.8939 x10^-81^ - NA | 0.993 | 4.01 | 55.11 | 17.00-338.18 | <0.001*** |
| PHI | 4.39 | 80.32 | 39.87-166.63 | <0.001*** | 2.622 | 13.76 | 1.36-103.69 | 0.0134 * | 3.19 | 24.34 | 11.41-51.85 | <0.001*** |
| allcrop | 0.74 | 2.09 | 1.77-2.48 | <0.001*** | 0.50 | 1.65 | 1.06-2.40 | 0.0125 * | 0.08 | 1.08 | 1.06-1.10 | <0.001*** |
| rnativeD | 0.15 | 1.16 | 1.12-1.21 | <0.001*** | 0.14 | 1.16 | 1.06-1.25 | <0.001*** | 0.13 | 1.14 | 1.09-1.19 | <0.001*** |
| popD |  |  |  |  |  |  |  |  |  |  |  |  |
| medium | 1.02 | 2.79 | 1.76-4.51 | <0.001*** | 0.97 | 2.63 | 0.56-18.46 | 0.249 | 0.60 | 1.82 | 1.09-3.08 | 0.02371 * . |
| high | -0.30 | 0.74 | 0.41-1.33 | 0.32 | -16.33 | 8.1150x10^-8^ | NA - 1.4421 x10^56^ | 0.991 | -1.78 | 0.17 | 0.05-0.44 | 0.00102 ** |
| butcherD | 0.88 | 2.40 | 1.58-3.59 | <0.001*** | -15.11 | 2.7538 x10^-8^ | NA - 3.1927 x10^64^ | 0.993 | 0.97 | 2.65 | 1.64-4.16 | <0.001*** |
| allrice | 0.14 | 1.15 | 1.02-1.28 | 0.01978 * | 0.40 | 1.49 | 1.01-2.20 | 0.038 * | 0.02 | 1.02 | 1.01-1.04 | <0.001*** |
| WetlandA | 0.14 | 1.15 | 1.01-1.30 | 0.02729 * | -0.07 | 0.93 | 0.33-1.50 | 0.836 | 0.01 | 1.01 | 0.99-1.02 | 0.185 |
| nrnwaterD | 0.06 | 1.06 | 1.03-1.09 | <0.001*** | 0.04 | 1.04 | 0.91-1.11 | 0.355 | 0.04 | 1.04 | 1.00-1.07 | 0.0148 * |
| rbroilerD | 0.15 | 1.16 | 1.07-1.25 | <0.001*** | 0.02 | 1.03 | 0.59-1.33 | 0.897 | 0.10 | 1.11 | 0.99-1.22 | 0.0507 . |
| rlayerD | 0.06 | 1.07 | 1.04-1.10 | <0.001*** | 0.04 | 1.04 | 0.98-1.08 | 0.0396 * | 0.04 | 1.04 | 1.02-1.07 | 0.00136 ** |

*p<0.05; **p<0.01, ***p<0.001

^#^OR: odds ratio

^$^CI: confidence interval
