# Supplementary material for: Ecological factors associated with persistent circulation of multiple highly pathogenic avian influenza viruses among poultry farms in Taiwan during 2015-17
Source: PLoS One. 2020 Aug 13;15(8):e0236581. doi: 10.1371/journal.pone.0236581 (PMC7425926; doi:10.1371/journal.pone.0236581)
Supplement: S2 Table — (DOCX) [file pone.0236581.s002.docx]

Table S2. Univariate logistic regression modeling results after stepwise selection comparing the hot zone and non-hot zone areas of HPAIV-confirmed outbreak farms based on 3km local spatial clustering analysis during two epidemic waves of 2015-2016 and 2017, Taiwan

|  | 2015-2016 | | | | 2017 | | | | |
| --- | --- | --- | --- | --- | --- | --- | --- | --- | --- |
|  | Estimate | OR^#^ | 95% CI^$^ | p-value | Estimate | OR | | 95% CI | p-value |
| nrwaterD |  |  |  |  |  | |  |  |  |
| medium | 2.69 | 14.70 | 5.11-48.03 | <0.001*** | 2.80 | | 16.52 | 5.97-49.70 | <0.001*** |
| high | 4.25 | 69.84 | 31.01-200.08 | <0.001*** | 3.42 | | 30.47 | 13.97-80.13 | <0.001*** |
| allrD |  |  |  |  |  | |  |  |  |
| medium | 2.62 | 13.74 | 3.84-87.60 | <0.001*** | 2.27 | | 9.66 | 2.57-62.70 | 0.00332 ** |
| high | 4.73 | 113.20 | 35.40-690.79 | <0.001*** | 4.01 | | 55.11 | 17.00-338.18 | <0.001*** |
| PHI | 4.25 | 70.10 | 34.92-144.54 | <0.001*** | 3.19 | | 24.34 | 11.41-51.85 | <0.001*** |
| allcrop | 0.55 | 1.73 | 1.37-2.18 | <0.001*** | 0.08 | | 1.08 | 1.06-1.10 | <0.001*** |
| rnativeD | 0.15 | 1.16 | 1.12-1.20 | <0.001*** | 0.13 | | 1.14 | 1.09-1.19 | <0.001*** |
| popD |  |  |  |  |  | |  |  |  |
| medium | 1.12 | 3.07 | 1.93-5.04 | <0.001*** | 0.60 | | 1.82 | 1.09-3.08 | 0.02371 * . |
| high | -0.38 | 0.68 | 0.36-1.26 | 0.23 | -1.78 | | 0.17 | 0.05-0.44 | 0.00102 ** |
| butcherD | 0.86 | 2.37 | 1.56-3.56 | <0.001*** | 0.97 | | 2.65 | 1.64-4.16 | <0.001*** |
| allrice | -0.01 | 0.99 | 0.84-1.14 | 0.854 | 0.02 | | 1.02 | 1.01-1.04 | <0.001*** |
| WetlandA | 0.14 | 1.15 | 1.01-1.30 | 0.0301 * | 0.01 | | 1.01 | 0.99-1.02 | 0.185 |
| nrnwaterD | 0.05 | 1.05 | 1.04-1.07 | <0.001*** | 0.04 | | 1.04 | 1.00-1.07 | 0.0148 * |
| rbroilerD | 0.14 | 1.15 | 1.06-1.25 | <0.001*** | 0.10 | | 1.11 | 0.99-1.22 | 0.0507 . |
| rlayerD | 0.07 | 1.07 | 1.04-1.11 | <0.001*** | 0.04 | | 1.04 | 1.02-1.07 | 0.00136 ** |

*p<0.05; **p<0.01, ***p<0.001

^#^OR: odds ratio

^$^CI: confidence interval
