# Supplementary material for: Ecological factors associated with persistent circulation of multiple highly pathogenic avian influenza viruses among poultry farms in Taiwan during 2015-17
Source: PLoS One. 2020 Aug 13;15(8):e0236581. doi: 10.1371/journal.pone.0236581 (PMC7425926; doi:10.1371/journal.pone.0236581)
Supplement: S3 Table — (DOCX) [file pone.0236581.s003.docx]

Table S3. Multivariate logistic regression modeling results after stepwise selection comparing the hot zone and non-hot zone areas of HPAIV-confirmed outbreak farms based on 3km local spatial clustering analysis during two epidemic waves of 2015-2016 and 2017, Taiwan

|  | 2015-2016 | | | | 2017 | | | |
| --- | --- | --- | --- | --- | --- | --- | --- | --- |
|  | Estimate | aOR^※^ | 95% CI | p-value | Estimate | aOR^※^ | 95% CI | p-value |
| nrwaterD |  |  |  |  |  |  |  |  |
| medium | 1.53 | 4.64 | 1.45-16.32 | 0.011* | 1.71 | 5.51 | 1.81-17.90 | <0.01** |
| high | 2.68 | 14.52 | 6.0-43.44 | <0.001*** | 2.22 | 9.17 | 3.73-26.20 | <0.001*** |
| allrD |  |  |  |  |  |  |  |  |
| medium | 1.90 | 6.67 | 1.75-43.90 | 0.015* | 1.62 | 5.07 | 1.28-33.74 | 0.04* |
| high | 3.16 | 23.58 | 6.85-148.53 | <0.001*** | 2.11 | 8.23 | 2.12-54.86 | 0.01** |
| PHI | 2.26 | 9.55 | 3.95-23.95 | <0.001*** | 1.02 | 2.79 | 1.00-7.69 | 0.05* |
| allcrop | 0.28 | 1.32 | 0.99-1.77 | 0.06 | 0.04 | 1.04 | 1.02-1.07 | <0.001*** |
| rnativeD |  |  |  |  | 0.04 | 1.04 | 0.99-1.10 | 0.14 |
| popD |  |  |  |  |  |  |  |  |
| medium | 0.38 | 1.46 | 0.78-2.81 | 0.24 | -0.43 | 0.65 | 0.35-1.21 | 0.18 |
| high | -0.50 | 0.61 | 0.27-1.33 | 0.22 | -2.02 | 0.13 | 0.04-0.37 | <0.001*** |
| butcherD | 0.39 | 1.48 | 0.86-2.55 | 0.15 |  |  |  |  |
| allrice | -0.22 | 0.80 | 0.66-0.96 | 0.02* |  |  |  |  |

※aOR: adjusted odds ratio

*p<0.05; **p<0.01, ***p<0.001
