# Supplementary material for: Ecological factors associated with persistent circulation of multiple highly pathogenic avian influenza viruses among poultry farms in Taiwan during 2015-17
Source: PLoS One. 2020 Aug 13;15(8):e0236581. doi: 10.1371/journal.pone.0236581 (PMC7425926; doi:10.1371/journal.pone.0236581)
Supplement: S4 Table — (DOCX) [file pone.0236581.s004.docx]

Table S4. Multivariate logistic regression modeling results after stepwise selection comparing the hot zone and non-hot zone areas of HPAIV-confirmed outbreak farms based on 3km local spatial clustering analysis of H5N3 and H5N6 in 2015 and 2017, respectively

|  | H5N3 (2015) | | | | H5N6 (2017) | | | |
| --- | --- | --- | --- | --- | --- | --- | --- | --- |
|  | Estimate | aOR^※^ | 95% CI | p-value | Estimate | aOR^※^ | 95% CI | p-value |
| nrwaterD |  |  |  |  |  |  |  |  |
| medium |  |  |  |  | 19.85 | 4.16*10^8^ | NA^§^ | 0.996 |
| high |  |  |  |  | 20.58 | 8.65*10^8^ | NA | 0.996 |
| allrD |  |  |  |  |  |  |  |  |
| medium |  |  |  |  | 19.52 | 3*10^9^ | NA | 0.997 |
| high |  |  |  |  | 16.67 | 1.73*10^7^ | NA | 0.997 |
| PHI | 4.85 | 127 | 11.7-211 | <0.001*** |  |  |  |  |
| rnativeD | 0.172 | 1.19 | 1.07-1.32 | <0.01** |  |  |  |  |
| rbroilerD  medium  high | 2.13  -14.04 | 8.41  7.95*10^-7^ | 1.55-47.8  NA | 0.01*  0.99 |  |  |  |  |
| rlayerD  medium  high | 2.71  2.86 | 15.1  17.5 | 1.69-448  1.89-478 | 0.04*  0.03* |  |  |  |  |
| popD |  |  |  |  |  |  |  |  |
| medium | 17.87 | 5.79*10^7^ | NA | 0.99 | -1.46 | 0.23 | 0.03-1.27 | 0.10 |
| high | 19.45 | 2.79*10^8^ | NA | 0.99 | -20.06 | 1.93*10^-9^ | NA | 0.997 |

※aOR: adjusted odds ratio

^§^NA: non-available due to large standard error

*p<0.05; **p<0.01, ***p<0.001
