# Supplementary material for: Ecological factors associated with persistent circulation of multiple highly pathogenic avian influenza viruses among poultry farms in Taiwan during 2015-17
Source: PLoS One. 2020 Aug 13;15(8):e0236581. doi: 10.1371/journal.pone.0236581 (PMC7425926; doi:10.1371/journal.pone.0236581)

Fig S1. Spatial autocorrelation (Global Moran’s I) of outbreak farms due to HPAI viruses by distance. A peak Z score at 3km suggests that spatial processes exist at this distance to produce pronounced spatial clustering.


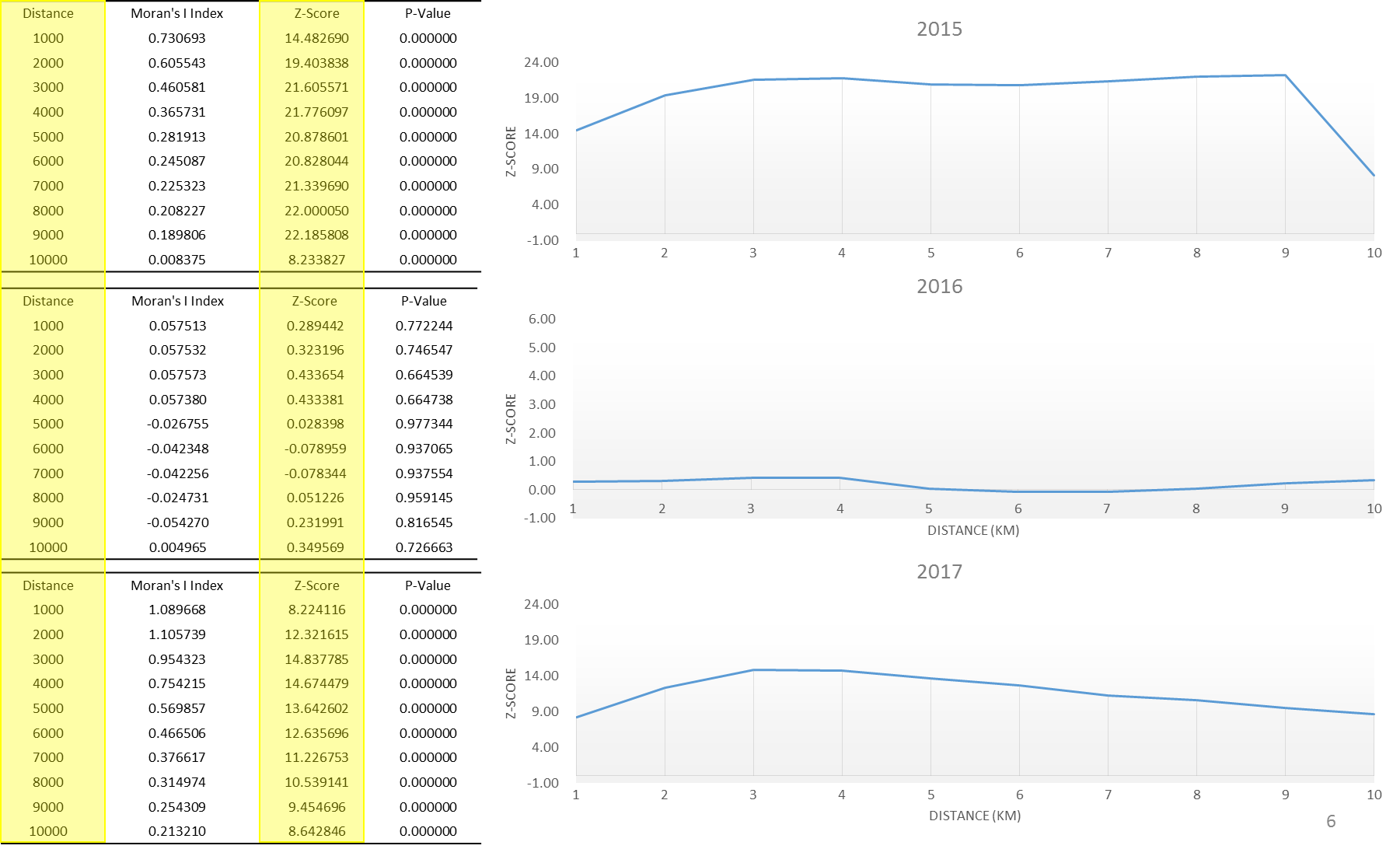

Supplement: S1 Fig — A peak Z score at 3km suggests that spatial processes exist at this distance to produce pronounced spatial clustering. (DOCX) [file pone.0236581.s005.docx]
