# Supplementary material for: Ecological factors associated with persistent circulation of multiple highly pathogenic avian influenza viruses among poultry farms in Taiwan during 2015-17
Source: PLoS One. 2020 Aug 13;15(8):e0236581. doi: 10.1371/journal.pone.0236581 (PMC7425926; doi:10.1371/journal.pone.0236581)

Fig S2. Plots of generalized additive model(GAM) with the identified hotspots as the dependent variable. (a)-(h) showed different predictors on HPAI hotspot areas. (a) All registered farm density (allrD) showed non-linear relationship with dependent variable. However, since the sparse data points at high farm density, allrD was trichotomized based on 33 and 67 percentile. (b) Non-registered non-waterfowl flock density (nrnwaterD) showed non-linear relationship with dependent variable and was trichotomized at 19 and 29. (c) Non-registered waterfowl flock density (nrwaterD) showed non-linear relationship with dependent variable. However, since the sparse data points at high farm density, allrD was trichotomized based on 33 and 67 percentile. (d) Registered broiler chicken farm density (rbroilerD) ) showed non-linear relationship with dependent variable and was trichotomized at 2, 12. (e) Registered layer chicken farm density (rlayerD) showed non-linear relationship with dependent variable. However, since the sparse data points at high farm density, allrD was trichotomized based on 33 and 67 percentile. (f) Registered native chicken farm density (rnativeD) showed linear relationship with dependent variable. (g) Butcher house density (rbutcherD) showed linear relationship with dependent variable. (h) Population density (popD104) ) showed non-linear relationship with dependent variable. However, since the sparse data points at high farm density, allrD was trichotomized based on 33 and 67 percentile.


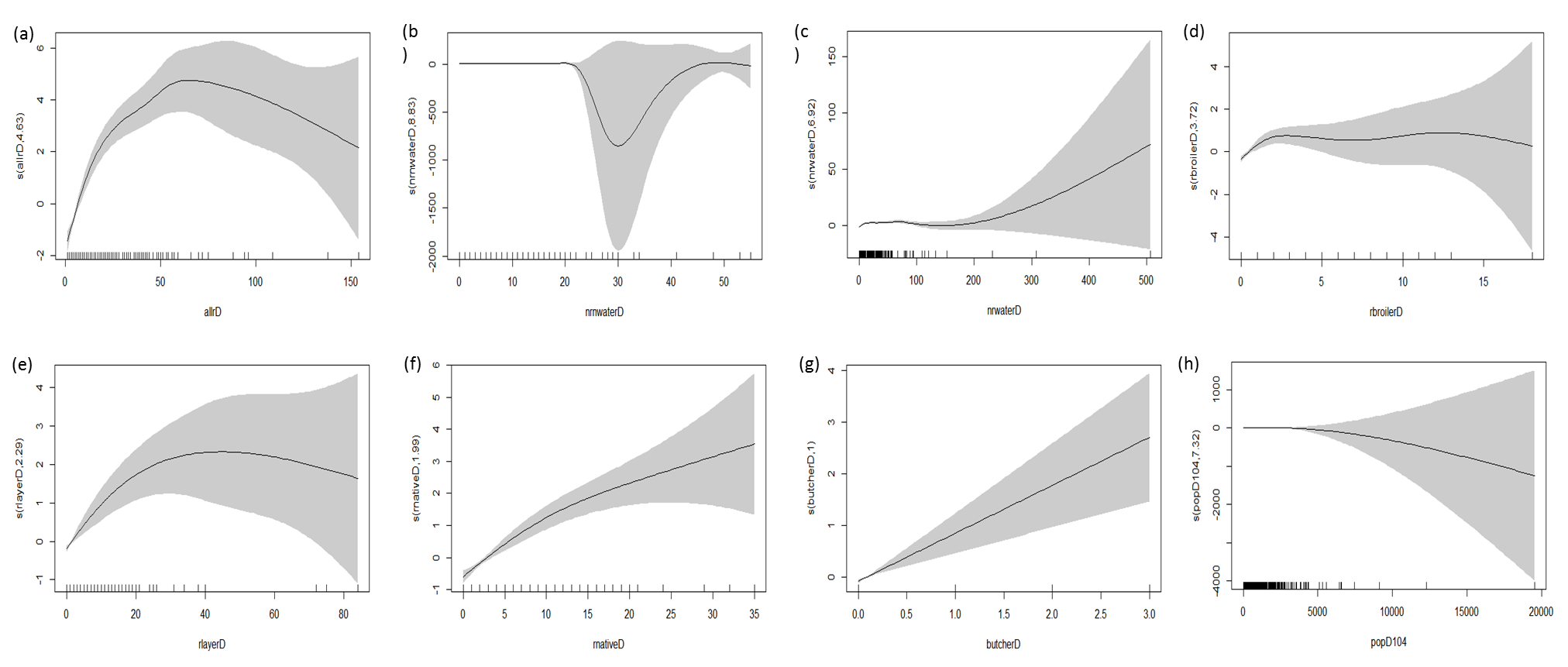

Supplement: S2 Fig — (a)-(h) showed different predictors on HPAI hotspot areas. (a) All registered farm density (allrD) showed non-linear relationship with dependent variable. However, since the sparse data points at high farm density, allrD was trichotomized based on 33 and 67 percentile. (b) Non-registered non-waterfowl flock density (nrnwaterD) showed non-linear relationship with dependent variable and was trichotomized at 19 and 29. (c) Non-registered waterfowl flock density (nrwaterD) showed non-linear relationship with dependent variable. However, since the sparse data points at high farm density, allrD was trichotomized based on 33 and 67 percentile. (d) Registered broiler chicken farm density (rbroilerD)) showed non-linear relationship with dependent variable and was trichotomized at 2, 12. (e) Registered layer chicken farm density (rlayerD) showed non-linear relationship with dependent variable. However, since the sparse data points at high farm density, allrD was trichotomized based on 33 and 67 percentile. (f) Registered native chicken farm density (rnativeD) showed linear relationship with dependent variable. (g) Butcher house density (rbutcherD) showed linear relationship with dependent variable. (h) Population density (popD104)) showed non-linear relationship with dependent variable. However, since the sparse data points at high farm density, allrD was trichotomized based on 33 and 67 percentile. (DOCX) [file pone.0236581.s006.docx]
