# Supplementary material for: Ecological factors associated with persistent circulation of multiple highly pathogenic avian influenza viruses among poultry farms in Taiwan during 2015-17
Source: PLoS One. 2020 Aug 13;15(8):e0236581. doi: 10.1371/journal.pone.0236581 (PMC7425926; doi:10.1371/journal.pone.0236581)

Fig S3. Temporal distribution of highly pathogenic avian influenza (HPAI) H5Nx in poultry farms by poultry types, (A) chicken, (B) duck, (C) goose, in Taiwan from 2015 to 2017.


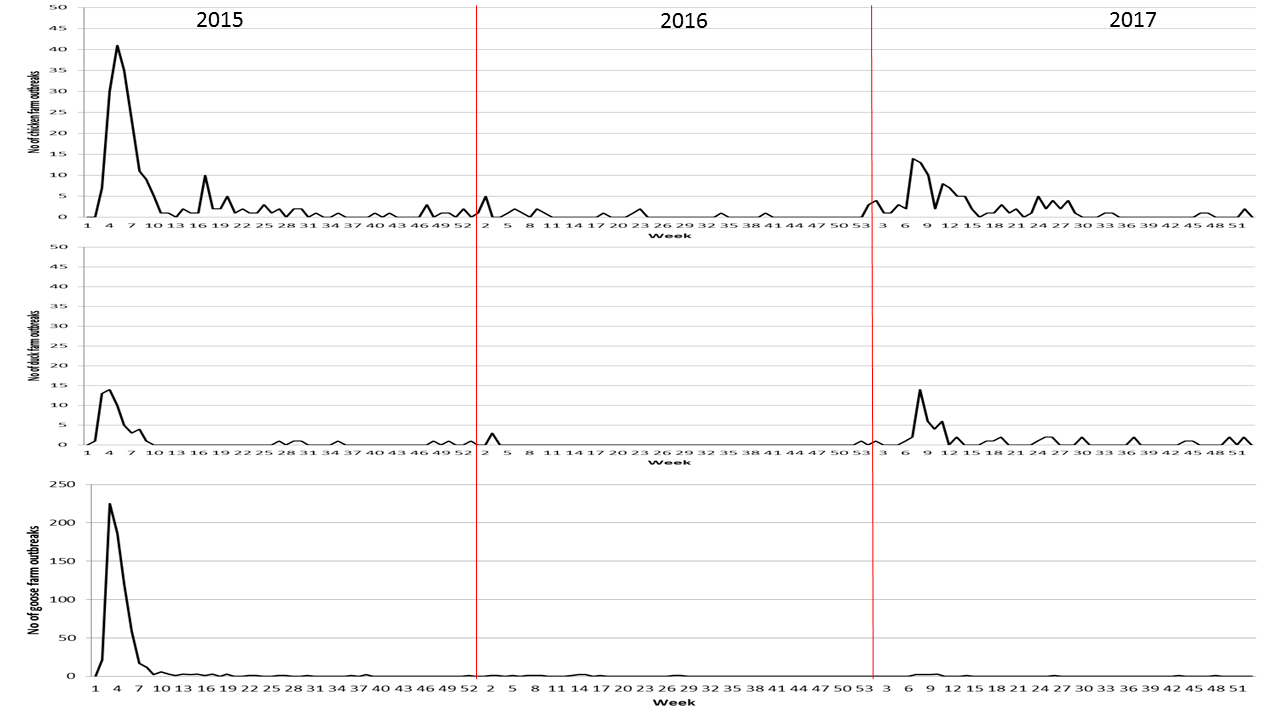

Supplement: S3 Fig — Temporal distribution of highly pathogenic avian influenza (HPAI) H5Nx in poultry farms by poultry types, (A) chicken, (B) duck, (C) goose, in Taiwan from 2015 to 2017. (DOCX) [file pone.0236581.s007.docx]
