# Supplementary material for: Ecological factors associated with persistent circulation of multiple highly pathogenic avian influenza viruses among poultry farms in Taiwan during 2015-17
Source: PLoS One. 2020 Aug 13;15(8):e0236581. doi: 10.1371/journal.pone.0236581 (PMC7425926; doi:10.1371/journal.pone.0236581)

Fig S4. Spatial distribution of HPAI hotspot outbreak farms by different HPAI subtypes under 3x3 km grid identified by Local Moran’s I spatial autocorrelation analyses during 2015. (a) The high-high (HH) hotspot areas for outbreak farms by H5N2 and H5N8; (b) the HH areas for outbreak farms by H5N3; (c) Merge of HH areas of H5N2/H5N8 and H5N3. The overlapping hotspots colored in yellow


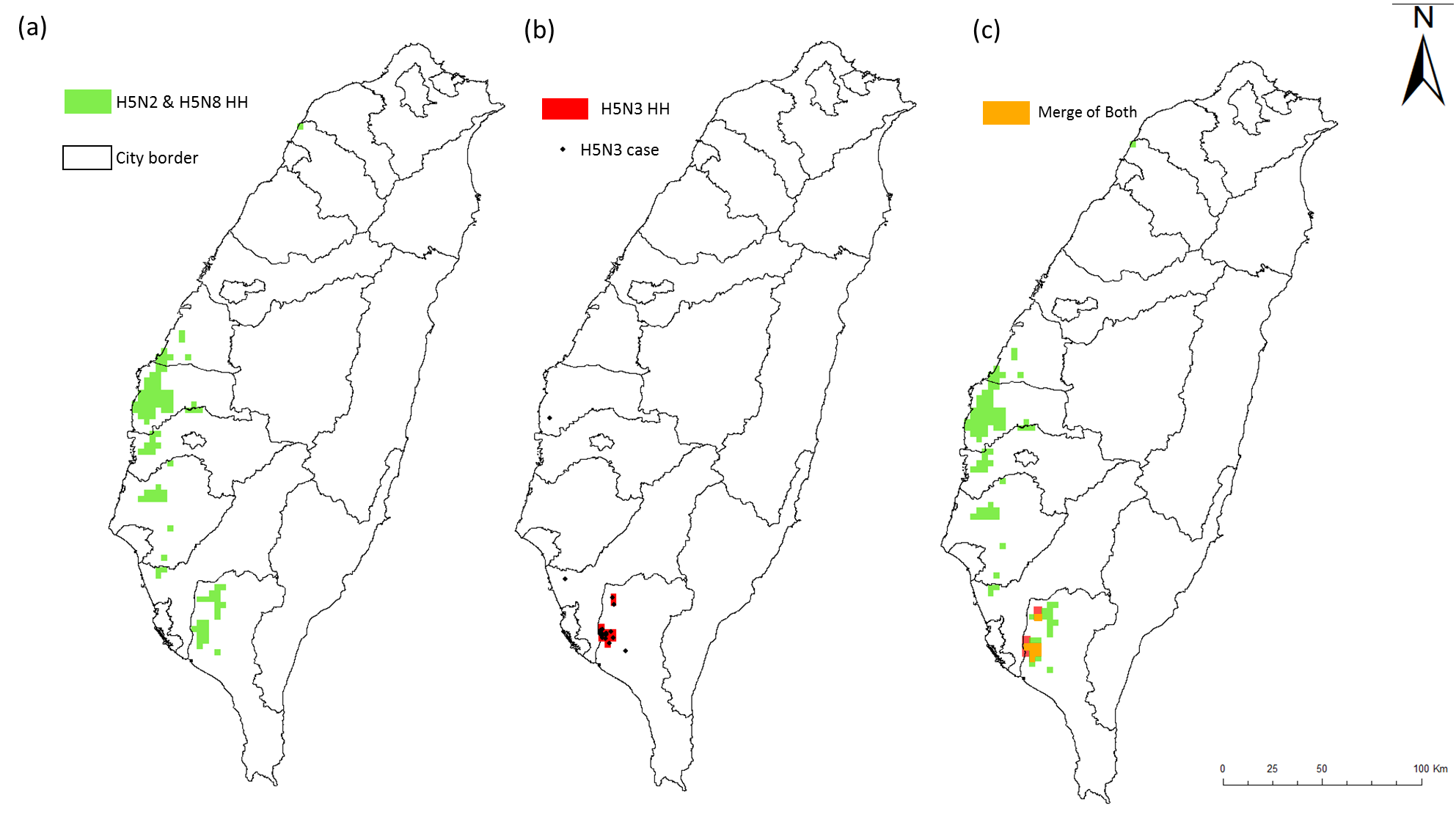

Supplement: S4 Fig — (a) The high-high (HH) hotspot areas for outbreak farms by H5N2 and H5N8; (b) the HH areas for outbreak farms by H5N3; (c) Merge of HH areas of H5N2/H5N8 and H5N3. The overlapping hotspots colored in yellow. (DOCX) [file pone.0236581.s008.docx]
