# Supplementary material for: Ecological factors associated with persistent circulation of multiple highly pathogenic avian influenza viruses among poultry farms in Taiwan during 2015-17
Source: PLoS One. 2020 Aug 13;15(8):e0236581. doi: 10.1371/journal.pone.0236581 (PMC7425926; doi:10.1371/journal.pone.0236581)

Fig S6. Molecular dating of the global dissemination of clade 2.3.4.4 influenza A viruses during 2014-2015. (A) Maximum likelihood phylogeny of clade 2.3.4.4 H5 viruses based on the hemagglutinin (HA) gene. The two clades containing viruses isolated during the first wave (2014-2015) and the second wave (2016-2017) of the global dispersal of H5Nx were identified and labeled. Within the 2014-2015 sublineage, the viruses isolated in North Asia (Korea, Japan and Russia), Europe, North America and Taiwan were colored as blue, purple, green and red, respectively. (B) Time-scaled HA phylogeny of viruses responsible for 2014-2015 global outbreaks. Branch colors indicate inferred ancestral geographical regions of each branch. Sequences identified from wild birds were highlighted with dark dots on the tips. (C) Posterior probability distributions of the time of the most recent common ancestor (tMRCA) for viruses isolated in distinct countries during 2014-2015. Internal nodes corresponding to the position of tMRCAs were illustrated with dashed lines.


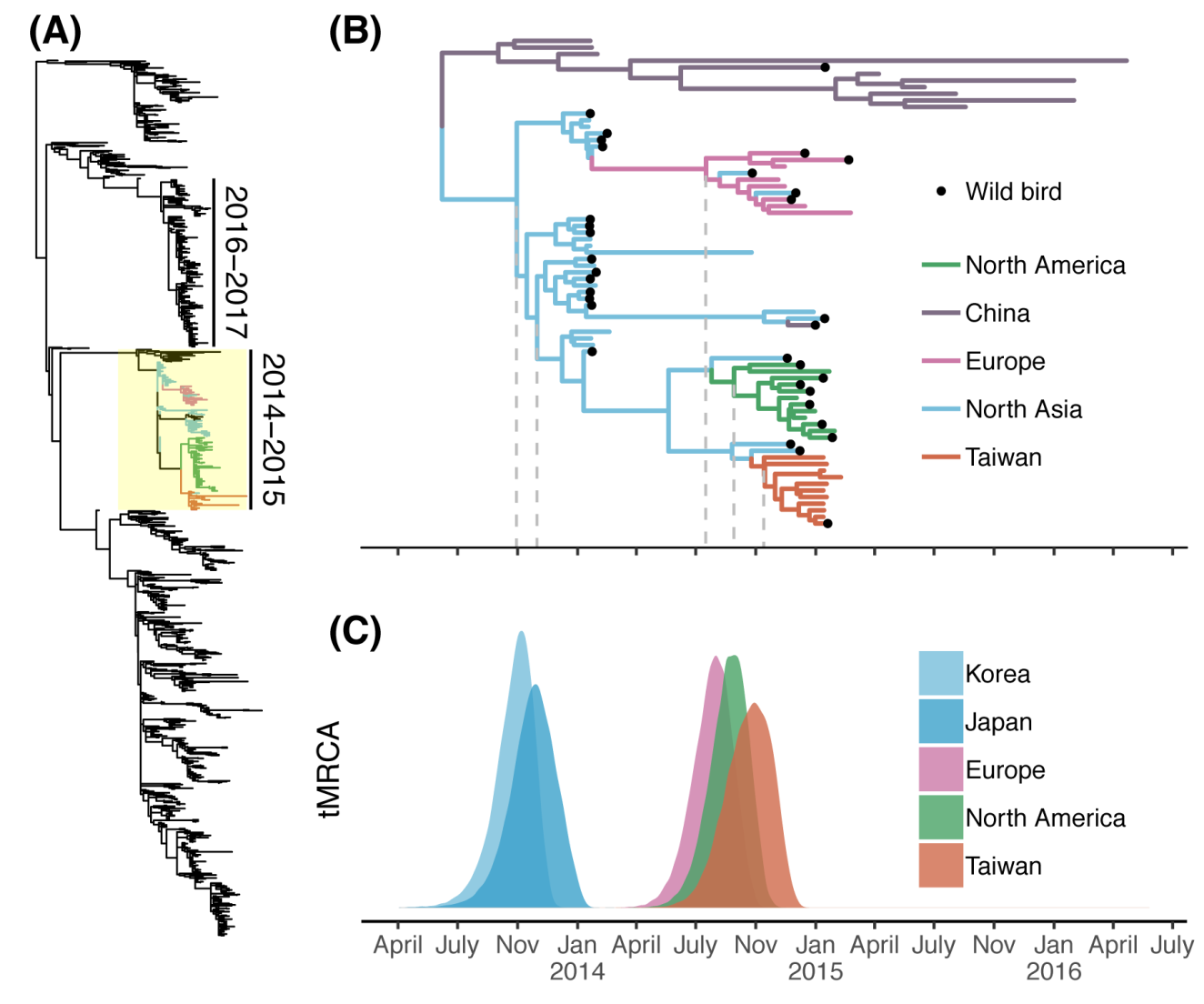

Supplement: S6 Fig — (A) Maximum likelihood phylogeny of clade 2.3.4.4 H5 viruses based on the hemagglutinin (HA) gene. The two clades containing viruses isolated during the first wave (2014–2015) and the second wave (2016–2017) of the global dispersal of H5Nx were identified and labeled. Within the 2014–2015 sublineage, the viruses isolated in North Asia (Korea, Japan and Russia), Europe, North America and Taiwan were colored as blue, purple, green and red, respectively. (B) Time-scaled HA phylogeny of viruses responsible for 2014–2015 global outbreaks. Branch colors indicate inferred ancestral geographical regions of each branch. Sequences identified from wild birds were highlighted with dark dots on the tips. (C) Posterior probability distributions of the time of the most recent common ancestor (tMRCA) for viruses isolated in distinct countries during 2014–2015. Internal nodes corresponding to the position of tMRCAs were illustrated with dashed lines. (DOCX) [file pone.0236581.s010.docx]
